# Clinical Significance of Epidurography Contrast Patterns after Adhesiolysis during Lumbar Percutaneous Epidural Neuroplasty

**DOI:** 10.1155/2018/6268045

**Published:** 2018-04-01

**Authors:** Sang-Hyuk Park, Gyu Yeul Ji, Pyung Goo Cho, Dong Ah Shin, Young Sul Yoon, Keung Nyun Kim, Chang Hyun Oh

**Affiliations:** ^1^Department of Neurosurgery, Yonsei Barun Hospital, Seoul, Republic of Korea; ^2^Department of Neurosurgery, Spine and Spinal Cord Research Institute, Yonsei University College of Medicine, Seoul, Republic of Korea; ^3^Department of Neurosurgery, Bundang Jesaeng General Hospital, Seongnam, Republic of Korea; ^4^Department of Neurosurgery, Cham Teun Teun Hospital, Guri, Republic of Korea

## Abstract

**Background:**

The correlation between epidurography contrast patterns and the clinical outcomes of percutaneous epidural neuroplasty (PEN) remains unclear.

**Objective:**

To analyze the correlation between postadhesiolysis epidurography contrast patterns and the clinical outcomes of patients who undergo lumbar PEN.

**Design:**

This study is a retrospective analysis of 78 consecutive patients who underwent lumbar PEN between April 2012 and March 2013.

**Setting:**

The analysis was done in the university hospital center.

**Method:**

The clinical outcomes of all patients were assessed before and 1, 3, 6, and 12 months after undergoing lumbar PEN. Specifically, the intensity of back and leg pain, quality of life, and procedural outcomes were evaluated using a visual analog scale (VAS), the Oswestry Disability Index (ODI), and the 12-Item Short-Form Health Survey (SF-12).

**Results:**

The VAS scores for back and leg pain, ODI score, and SF-12 score exhibited a significant improvement during the follow-up period (*P* < 0.01 versus preprocedural scores). At most follow-up time points, patients exhibiting extraforaminal contrast distribution (*n*=22) on postadhesiolysis epidurograms exhibited a similar improvement in VAS scores and a significantly better improvement in ODI and SF-12 scores compared with patients exhibiting intracanal contrast distribution (*n*=56).

**Conclusion:**

Extraforaminal contrast distribution during lumbar PEN may be associated with better functional outcomes.

## 1. Introduction

Chronic low back pain is a common health problem with widespread socioeconomical reverberations which is experienced by most individuals at some point in their lives [1–3]. Several studies on low back pain have been conducted, and many treatments have been applied, with most physicians focusing on lumbar disc herniation as a leading cause for spinal interventions [4–7]. The demand for spinal interventions is increasing because of their minimal invasiveness and therapeutic efficacy [4–7]. Percutaneous epidural neuroplasty (PEN) is a novel, widely used technique for the lysis of microscopic adhesions surrounding nerve tissues and delivery of therapeutic drugs directly to the target area [8–15]. However, spinal interventions may not always be successful because of several barriers. For example, postoperative scarring inhibits epidural contrast spread, while foraminal stenosis blocks the extraforaminal extension of injected drugs [16, 17]. In addition to obvious masses and canal narrowing, microscopic adhesions, which may be a result of chronic inflammation, can interrupt the spread of drugs. Although numerous studies have described PEN procedures, only a few have reported intraoperative findings that indicate the surgical outcomes [11, 13, 14, 17–19]. Epidurography is always performed during PEN procedures, but there is insufficient evidence supporting the usefulness of this procedure. Moreover, the correlation between epidurography contrast patterns and the clinical outcomes of PEN has not been frequently reported [19, 20]. Postadhesiolysis epidurography patterns are assumed to be indicators of surgical outcomes. In addition, the presence of poor contrast spread on postadhesiolysis epidurograms may be associated with the requirement for repeat adhesiolysis procedures or a change in the target area for achieving better contrast patterns. In the present study, we assessed the clinical outcomes of lumbar PEN on the basis of contrast patterns on postadhesiolysis epidurograms.

## 2. Methods

### 2.1. Patient Selection

This retrospective analysis of medical and radiographic records included 78 patients who underwent lumbar PEN procedures between April 2012 and March 2013 at a single university hospital. The study protocol was approved by the ethics committee, and informed consent was obtained from all subjects. The inclusion criterion was chronic low back pain with or without leg pain due to lumbar disc herniation and/or lumbar spinal stenosis. All patients reported a history of discogenic or radicular symptoms refractory to conservative treatments for a minimum of 6 weeks. Diagnoses were established using magnetic resonance imaging (MRI) and/or computed tomography (CT) performed before PEN. Patients with a history of spinal surgery and those with cauda equina syndrome, bleeding diathesis, associated somatic or psychiatric disease, vertebral fractures, pregnancy, and tumors or other underlying systemic diseases that could significantly affect the procedural outcomes were excluded. All procedures were performed by one of the authors (DAS) using the same procedural protocol.

### 2.2. PEN Procedure

A standard PEN procedure was used to lyse adhesions and achieve nerve blockades in all patients, as previously described [11, 14]. A 1-day protocol was followed. The patient was placed on a radiolucent table in the prone position, and the procedure was performed under fluoroscopic guidance. The coccygeal and sacral regions were disinfected with 10% Betadine, and the surgical site was draped in the usual aseptic manner. The sacral hiatus was anesthetized with 1% xylocaine. Then, a 20-G Tuohy needle was introduced into the epidural space below the level of S3. A total of 3 mL of the contrast agent (Omnipaque, GE Healthcare Korea, Seoul, Republic of Korea) was instilled to confirm the epidural space and preadhesiolysis status. Both anteroposterior and lateral fluoroscopic views were obtained. We also assessed the development of any adverse reactions. On confirmation of the target for PEN, a catheter specialized for adhesiolysis (TUN-L-KATH; Epimed, TX, United States) was gently inserted toward the target site. For better catheter manipulation, the tip was bent for an approximate length of 1 cm. The catheter was easily navigated by rotation. Once it reached the target site, a second epidurogram was obtained by injecting 3 mL of the contrast agent for the identification of filling defects or cutoff signs surrounding the target area. When the tip of the catheter touched the target site or the contrast agent exerted pressure on the lesion, patients were asked to report provoked symptoms. According to the surgical records, they frequently reported pain similar to what they had been suffering. Both mechanical and chemical adhesiolysis were performed. The former was achieved through pushing, pulling, and rotating movements of the catheter, while the latter was achieved by the injection of a mixture comprising 0.9% normal saline (10 mL) and 3000 U of hyaluronidase (H-lase; 1500 U/mL; Kuhnil, South Korea). Following adhesiolysis, a third epidurogram was obtained using 3 mL of the contrast agent. The patterns of contrast dispersal were examined on both anteroposterior and lateral fluoroscopic views. All epidurogram images were saved in the Digital Imaging and Communications in Medicine format for future analysis, and the third epidurogram was used for analysis in our study. Finally, a mixture of 0.2% ropivacaine (8 mL; Naropin; Astrazeneca Korea, Seoul, Republic of Korea) and 40% triamcinolone acetonide (20 mg; triamcinolone; DongKwang, Seoul, Republic of Korea) was slowly injected. The stored movie clips and still images were independently reviewed twice by two authors who were blinded to the patient data (SHP and PGC).

### 2.3. Epidurography Contrast Patterns

**Figure 1:**
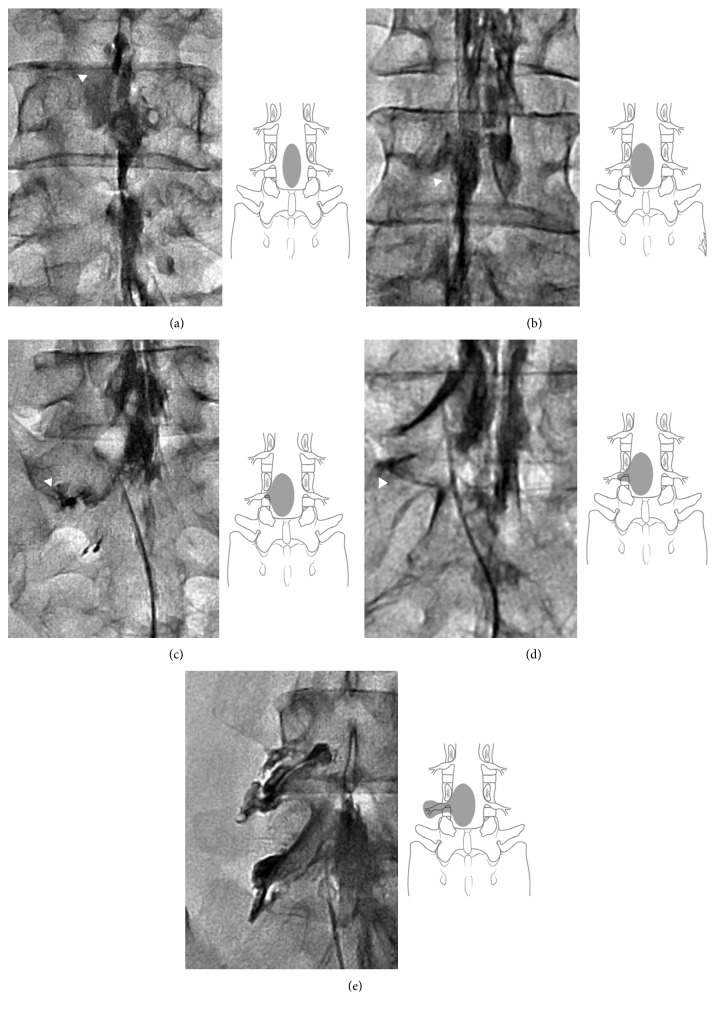
Modified classification of epidurography contrast patterns. (a) Grade 1: contrast spread to the medial or midline zone of the ipsilateral or contralateral epidural space, (b) grade 2: contrast spread to the lateral epidural space, proximal to the medial border of the neural foramen, (c) grade 3: contrast spread to the intraforaminal space, not extending to the lateral border of the neural foramen, (d) grade 4: contrast spread to the intraforaminal space, extending to but not crossing the lateral border of the neural foramen, and (e) grade 5: contrast spread beyond the lateral border of the neural foramen. Patients with grade 1, grade 2, and grade 3 patterns were assigned to a group exhibiting limited intracanal spread (IC group), while patients with grade 4 and grade 5 patterns were assigned to a group exhibiting extended extraforaminal spread (EF group).

Postadhesiolysis epidurography contrast patterns were defined and classified into five grades according to the system proposed by Mathis et al. [21]: grade 1, contrast spread to the medial or midline zone of the ipsilateral or contralateral epidural space; grade 2, contrast spread to the lateral epidural space, proximal to the medial border of the neural foramen; grade 3, contrast spread to the intraforaminal space, not extending to the lateral border of the neural foramen; grade 4, contrast spread to the intraforaminal space, extending to but not crossing the lateral border of the neural foramen; and grade 5, contrast spread beyond the lateral border of the neural foramen (Figure 1). For the comparison of clinical outcomes according to the contrast distribution pattern on epidurograms, patients with grade 1, grade 2, and grade 3 patterns were assigned to a group exhibiting limited intracanal spread (IC group), while patients with grade 4 and grade 5 patterns were assigned to a group exhibiting extended extraforaminal spread (EF group).

### 2.4. Follow-Up and Assessment of Surgical Outcomes

All patients were clinically evaluated before and 1, 3, 6, and 12 months after PEN by a nurse specialized in pain management and blinded to the treatment details. The intensity of leg and back pain was assessed using a subjective visual analog scale (VAS) calibrated from 0 to 10 (0 = no pain and 10 = the worst pain imaginable). For functional assessments, the Korean versions of the Oswestry Disability Index (ODI) and 12-Item Short-Form Health Survey (SF-12) were used [22, 23]. ODI evaluated the clinical effectiveness of PEN in terms of pain reduction and functional improvement, with the score ranging from 0 to 50. SF-12 incorporates two dimensions: a physical component summary (PCS) and a mental component summary (MCS). PCS and MCS scores are computed from the scores for 12 questions and range from 0 to 100, with a higher score representing better physical or mental health [19].

### 2.5. Statistical Analysis

Age, the duration of symptoms and follow-up, and VAS, ODI, and SF-12 scores are expressed as means ± standard deviations. Student's *t*-tests were used to assess differences in VAS, ODI, and SF-12 scores at each time point between the EF and IC groups. Chi-square tests were used to compare clinical outcomes between the two groups. Spearman's rank correlation tests were used to determine the correlation between epidurography contrast patterns and clinical outcomes. All statistical analyses were performed using the Statistical Package for the Social Sciences software (SPSS Inc., Chicago, Illinois, USA). A *P* value of <0.05 was considered statistically significant.

## 3. Results

### 3.1. Patient Characteristics

A total of 78 patients (27 men and 51 women) were included in this study. All patients were clinically followed up for more than 11 months. The mean age was 58.1 ± 14.1 years (range, 18 to 84 years), and the average duration of major symptoms before PEN was 7.1 ± 4.1 months (range, 2 to 31 months). Before PEN, the VAS score for back pain, VAS score for leg pain, ODI score, and SF-12 score were 5.8 ± 2.6, 5.0 ± 3.1, 19.4 ± 7.0, and 30.0 ± 7.2, respectively (Table 1).

### 3.2. Treatment Outcomes

**Figure 2:**
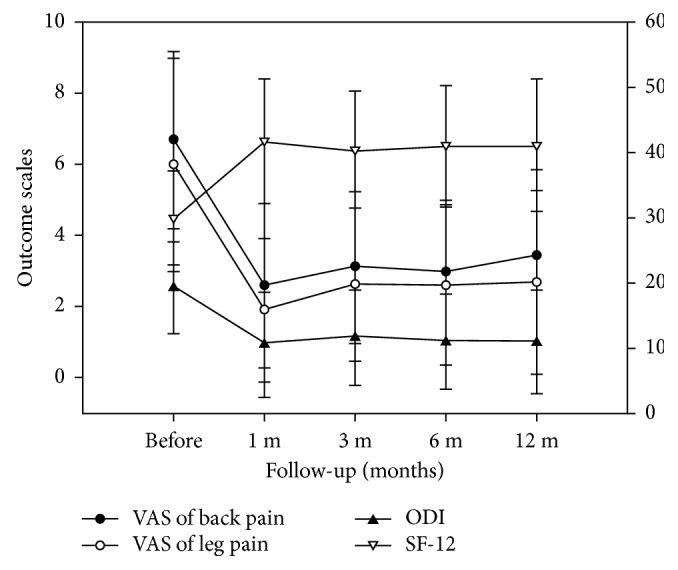
Clinical outcomes of patients who underwent lumbar PEN. VAS, ODI, and SF-12 scores before and 1, 3, 6, and 12 months after lumbar PEN. All postprocedural clinical scores have significantly improved compared with preprocedural scores (*P* < 0.001). VAS: visual analog scale, ODI: Oswestry Disability Index, SF-12: 12-Item Short-Form Health Survey, and PEN: percutaneous epidural neuroplasty.

The clinical outcomes of patients exhibited a significant improvement during the follow-up period. At 1, 3, 6, and 12 months after PEN, the VAS score for back pain was 2.6 ± 2.3, 3.1 ± 2.1, 3.0 ± 2.0, and 3.4 ± 2.4, respectively; the VAS score for leg pain was 1.9 ± 2.0, 2.6 ± 2.1, 2.6 ± 2.2, and 2.7 ± 2.6, respectively; the ODI score was 10.6 ± 8.0, 11.7 ± 7.4, 11.0 ± 7.3, and 11.0 ± 8.0, respectively; and the SF-12 score was 41.8 ± 9.6, 40.5 ± 9.0, 41.2 ± 9.2, and 41.2 ± 10.2, respectively (*P* < 0.01 when compared with preprocedural scores; Figure 2).

### 3.3. Postadhesiolysis Epidurography Contrast Patterns and Clinical Outcomes

**Figure 3:**
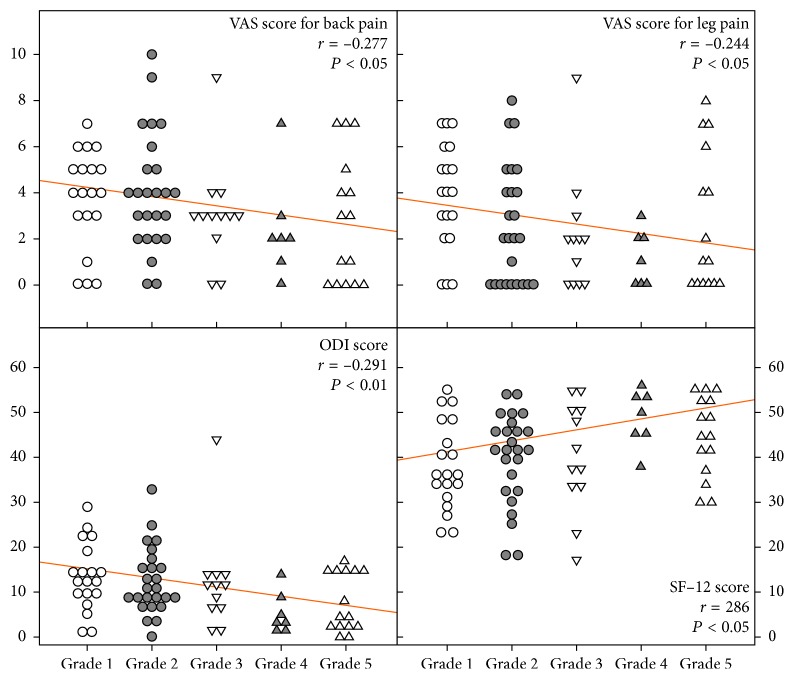
Correlation between epidurography contrast spread after adhesiolysis during PEN and clinical outcomes. Extraforaminal contrast spread (grades 4 and 5) is associated with a tendency for decreased pain and significantly better quality of life compared with intracanal spread (grades 1, 2, and 3). PEN: percutaneous epidural neuroplasty, VAS: visual analog scale, ODI: Oswestry Disability Index, and SF-12: 12-Item Short-Form Health Survey.

**Figure 4:**
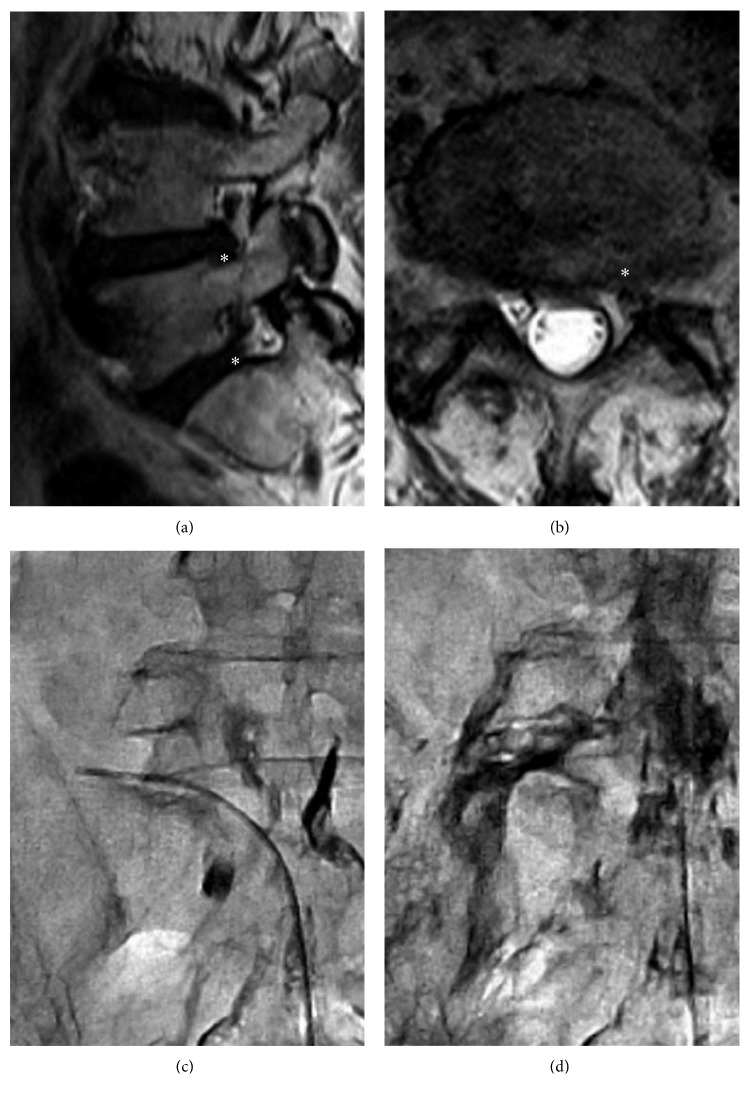
Representative case of a 60-year-old man with radicular pain in the left leg who underwent lumbar PEN. (a, b) Magnetic resonance imaging shows lumbar disc herniation and foraminal stenosis at the level of L4-5-S1 (asterisks). (c) The first epidurogram shows a filling defect at the left L5-S1 foramen. The catheter is inserted to the stenotic foramen, and mechanical adhesiolysis is attempted. (d) The final postadhesiolysis epidurogram shows excellent extraforaminal contrast spread. The patient exhibited significant pain relief and functional recovery after PEN. PEN: percutaneous epidural neuroplasty.

The patients were categorized into IC and EF groups according to the contrast patterns on postadhesiolysis epidurograms. In the IC group (*n*=56), there were 19, 25, and 12 patients with grade 1, grade 2, and grade 3 patterns, respectively. In the EF group (*n*=22), there were 7 and 15 patients with grade 4 and grade 5 patterns, respectively. Before PEN, the VAS score for back pain was 5.7 ± 2.6 and 6.0 ± 2.4 (*P*=0.639), the VAS score for leg pain was 4.7 ± 3.1 and 6.0 ± 2.8 (*P*=0.101), the ODI score was 20.0 ± 6.9 and 17.8 ± 7.1 (*P*=0.222), and the SF-12 score was 29.6 ± 7.0 and 31.0 ± 7.7 (*P*=0.452) in the IC and EF groups, respectively, with no significant differences between groups. At most follow-up time points, the EF group exhibited a similar improvement in VAS scores and a significantly better improvement in ODI and SF-12 scores compared with the IC group (Table 2). Thus, extraforaminal contrast spread was associated with a tendency for decreased pain and significantly better quality of life (Figure 3; Spearman's rank correlation test (*P* < 0.05)). A representative case of a patient with radicular pain in the left leg who exhibited significant pain relief and functional recovery after PEN is depicted in Figure 4.

## 4. Discussion

Epidurograms provide anatomical information and help in confirming accurate needle placement. Spinal interventions can be safely performed using a fluoroscope and epidurograms with radiopaque dye. The aim of the present study was to evaluate the correlation between contrast patterns on postadhesiolysis epidurograms and clinical outcomes in patients who underwent lumbar PEN. We observed that PEN ameliorated the pain associated with lumbar disc herniation and/or lumbar spinal stenosis. VAS scores for leg and back pain, ODI scores, and SF-12 scores exhibited a significant improvement over the entire 12-month follow-up period. Racz et al. [24] reported similar results in their study, where immediate pain relief was observed after PEN in more than 70% patients and the outcomes were maintained for 6 months. The success rate for PEN is reported to be 50%–71% [11, 14, 18, 25, 26]. The present study also showed good clinical outcomes, with the greatest improvement observed at 1 month after PEN. Moreover, the improved outcomes were maintained without much variation for 1 year after the procedure. The immediate effect of PEN is believed to result from the combined effects of mechanical adhesiolysis, chemical adhesiolysis, and local lavage of inflammatory mediators, which induce desensitization, neuromodulation, and local anesthesia [8, 27].

In the present study, we found that the clinical outcomes of lumbar PEN were correlated with the presence of extraforaminal contrast spread on the final epidurogram obtained after adhesiolysis. Extraforaminal contrast spread is believed to be an indicator of successful adhesiolysis. The purpose of PEN is to eliminate the barriers in the epidural space that disturb drug delivery. Extraforaminal contrast spread may be related to the creation of channels around the target site through adhesiolysis, and it facilitates drug delivery to the lesion site. Exposure to the ideal amount of medication can result in better pain reduction. It can also enhance the recovery of the perineural circulation, which can improve neural congestion and decrease the associated pain. The results of our study confirmed that access to the exact position of the lesion site is associated with a better outcome. These findings suggest that mechanical adhesiolysis is meaningful only at the lesion site. Moreover, if the exact lesion site cannot be reached, both adhesiolysis and drug delivery will fail and the target area will remain untreated.

In 1991, Kuslich et al. [28] demonstrated pain-sensitive structures in the spinal canal using mechanical and electrical stimulation. These structures include the annulus, nerve roots, posterior longitudinal ligament, facet joints, tendons, ligaments, and fascia [29]. There is evidence suggesting that the distribution of pain generators is increased in the ventral lateral space [29, 30]. Accordingly, we expected that placement of the catheter in the ventral epidural space would be strongly correlated with better outcomes. However, we could not confirm a direct correlation between ventral placement of the catheter and improved outcomes. Instead, we observed that ventral placement of the catheter tip was correlated more with the possibility of extraforaminal contrast spread, which resulted in better outcomes. It is assumed that adhesiolysis, rather than ventral catheter placement, affects clinical outcomes. Further studies with larger patient samples are necessary to clarify the direct effects of ventral catheter placement. We also found that the epidurography contrast pattern showed no significant differences among the five grades. Therefore, we suggest that division of the pattern into two grades may be adequate for evaluating the surgical outcomes of PEN.

In the present study, there were no direct correlations between cutoff signs or filling defects and the clinical outcomes of PEN. In our practice, we cannot easily resolve cutoff signs and filling defects. Cutoff signs are a result of severe spinal stenosis, while filling defects are frequently associated with disc herniations. Currently, gross adhesions cannot be easily lysed by soft PEN. However, regardless of the presence of cutoff signs or filling defects, better outcomes were achieved with extraforaminal contrast spread. There may be microscopic adhesions that are more relevant and can be lysed by soft PEN. Park et al. also reported that there was no correlation between the degree of pain relief and epidural filling defects in patients with lumbar stenosis [31].

This study has several limitations. These include the small sample size, the unequal groups, the short follow-up duration, and the retrospective design. Future studies should ideally include an appropriate control group. Nevertheless, the strength of our study is the use of clinical outcome measures, namely, ODI and SF-12, for assessment of the quality of life in addition to pain scores. Furthermore, the results of the study are more in line with the prognosis because the patients received a single treatment rather than repetitive treatments. With the use of epidurograms, physicians can design and plan adhesiolysis that aims at a precise target point and creates effective tunnels, which will help in predicting the prognosis.

## 5. Conclusions

In conclusion, the findings of our study suggest that the contrast patterns on final epidurograms obtained after adhesiolysis during PEN are indicators of clinical outcomes. Specifically, extraforaminal contrast spread during PEN is associated with better functional outcomes.

## Figures and Tables

**Table 1:** Baseline characteristics of patients with chronic low back pain with or without leg pain who underwent lumbar percutaneous epidural neuroplasty.

		Variable	Range
Number of patients (men : women)		78	27 : 51
Age (years)		58.1 ± 14.1	18–84
Symptom duration (months)		7.1 ± 4.1	2–31
Follow-up duration (months)		**12.7 ± 1.0**	11–16
VAS score	Back pain	5.8 ± 2.6	—
	Leg pain	5.0 ± 3.1	—
ODI score		19.4 ± 7.0	—
SF-12 score		30.0 ± 7.2	—

VAS: visual analog scale; ODI: Oswestry Disability Index; SF-12: 12-Item Short-Form Health Survey.

**Table 2:** Clinical outcomes of patients with limited intracanal contrast spread (IC group) and those with extended extraforaminal contrast spread (EC group) on postadhesiolysis epidurograms obtained during lumbar percutaneous epidural neuroplasty (PEN).

		VAS score for back pain	VAS score for leg pain	ODI score	SF-12 score
Before PEN	IC group	5.7 ± 2.6	4.7 ± 3.1	20.0 ± 6.9	29.6 ± 7.0
	EF group	6.0 ± 2.4	6.0 ± 2.8	17.8 ± 7.1	31.0 ± 7.7
	*P* value	0.639	0.101	0.222	0.452
1 month after PEN	IC group	2.9 ± 2.3	2.0 ± 2.1	12.1 ± 8.4	40.3 ± 10.2
	EF group	1.9 ± 2.1	1.7 ± 1.8	6.7 ± 5.6	45.7 ± 8.5
	*P* value	0.076	0.532	**0.007**	**0.023**
3 months after PEN	IC group	3.3 ± 2.2	2.6 ± 2.2	13.0 ± 7.6	39.4 ± 9.6
	EF group	2.7 ± 2.1	2.6 ± 1.9	8.5 ± 5.5	43.4 ± 8.7
	*P* value	0.317	0.924	**0.015**	**0.043**
6 months after PEN	IC group	3.3 ± 2.0	2.7 ± 2.3	12.6 ± 7.6	39.4 ± 9.4
	EF group	2.2 ± 2.0	2.2 ± 2.1	7.1 ± 4.8	46.0 ± 6.9
	*P* value	**0.032**	0.372	**0.002**	**0.007**
12 months after PEN	IC group	3.7 ± 2.4	2.9 ± 2.5	12.6 ± 8.2	39.3 ± 10.3
	EF group	2.7 ± 2.6	2.2 ± 2.7	7.0 ± 6.0	46.1 ± 8.3
	*P* value	0.087	0.274	**0.005**	**0.007**

VAS: visual analog scale; ODI: Oswestry Disability Index; SF-12: 12-Item Short-Form Health Survey.
